# Dynamic evolution of bone marrow adipocyte in B cell acute lymphoblastic leukemia: insights from diagnosis to post-chemotherapy

**DOI:** 10.1080/15384047.2024.2323765

**Published:** 2024-03-11

**Authors:** Xi Jia, Naying Liao, Yunqian Yao, Xutao Guo, Kai Chen, Pengcheng Shi

**Affiliations:** a Department of Hematology, Nanfang Hospital, Southern Medical University, Guangzhou, Guangdong, P. R. China; b Department of Radiotherapy, Cancer Hospital of Shantou University Medical College, Shantou, Guangdong, China

**Keywords:** Acute, lymphoblastic, leukemia, bone, marrow, microenvironment, adipocytes, patient-derived xenograft model

## Abstract

Adipocyte is a unique and versatile component of bone marrow microenvironment (BMM). However, the dynamic evolution of Bone Marrow (BM) adipocytes from the diagnosis of B cell Acute Lymphoblastic Leukemia (B-ALL) to the post-treatment state, and how they affect the progression of leukemia, remains inadequately explicated. Primary patient-derived xenograft models (PDXs) and stromal cell co-culture system are employed in this study. We show that the dynamic evolution of BM adipocytes from initial diagnosis of B-ALL to the post-chemotherapy phase, transitioning from cellular depletion in the initial leukemia niche to a fully restored state upon remission. Increased BM adipocytes retards engraftment of B-ALL cells in PDX models and inhibits cells growth of B-ALL in vitro. Mechanistically, the proliferation arrest of B-ALL cells in the context of adipocytes-enrichment niche, might attribute to the presence of adiponectin secreted by adipocytes themselves and the absence of cytokines secreted by mesenchymal stem cell (MSCs). In summary, our findings offer a novel perspective for further in-depth understanding of the dynamic balance between BMM and B-ALL.

## Introduction

B cell Acute Lymphoblastic Leukemia (B-ALL) is characterized by the arrested differentiation and clonal proliferation of B-lymphoid progenitor cells. While intrinsic genetic mutations within hematopoietic stem and progenitor cells are the primary drivers of B-ALL,^1^ mounting evidence underscores the pivotal role played by the bone marrow microenvironment (BMM) in the development, progression, and treatment resistance of B-ALL.^2–4^ Leukemia cells can hijack and destroy the normal BMM niche by disrupting crosstalk between leukemia cells and BM stroma cells, leading to an aberrant BMM reconstitution primarily presented with remolding in the quantity or function of constitutive BMM cells. Consequently, this facilitates the rapid expansion of leukemic cells, ultimately contributing to leukemogenesis and the development of chemoresistance.^5–7^ In addition, upon exposure to chemotherapeutic stress, BMMs also undergo further adaptation.^8,9^

The BMM is composed of various cell types, including osteoblasts, endothelial cells, perivascular reticular cells, adipocytes, and mesenchymal stem or stromal cells (MSCs). MSCs have long been recognized as a major component of the BMM, promoting leukemia cell proliferation and chemoresistance.^10,11^ Conversely, adipocytes, a distinctive and versatile component of the BMM, have only recently gained prominence after being overlooked for a significant period.^12^ Accumulating evidence suggests that adipocytes also influence the proliferation of leukemic blasts.^13,14^ However, their influence on leukemia blasts remains a subject of debate. Previous studies have shown that BM adipocytes are essential for the growth of leukemic cells during in vitro co-culture and shield them from chemotherapy exposure, through both contact- or cytokine-dependent mechanisms.^15–17^ Recent advances in our understanding of the BMM have indicated that BM adipocytes inhibit the development of ALL.^7,18^ Moreover, very few studies have explicitly explored the dynamic evolution of BM adipocytes from the initial stages of B-ALL to the post-treatment phase.

In this study, we demonstrate that BM adipocytes undergo dynamic changes during B-ALL outset and post-treatment phases, transitioning from cellular depletion in the initial leukemia niche to a fully restored state upon remission. An increased proportion of BM adipocytes hampers engraftment of B-ALL cells in patient-derived xenografts (PDX) models. Our functional studies reveal that adipocytes are unable to support the growth of B-ALL cells in vitro, and this effect may be attributed to the presence of adiponectin secreted by the adipocytes themselves and the absence of cytokines secreted by MSCs.

## Materials and methods

### Patient samples

All primary samples were collected at Nanfang Hospital with informed consent for research purposes. BM and peripheral blood samples were obtained from B-ALL patients at the time of their initial diagnosis and post-treatment, based on cell morphology (FAB classification), cytogenetics, and routine immunophenotyping. BM samples from five donors for hematopoietic stem cell transplantation were used as healthy controls. All related procedures were conducted under the approval of the Institutional Review Boards of Nanfang Hospital. Mononuclear cells were separated via density gradient centrifugation (Lymphoprep). The samples were cryopreserved in liquid nitrogen using RPMI 1640 supplemented with 40% fetal bovine serum (FBS) and 10% dimethyl sulfoxide (DMSO) or were directly transplanted into immunodeficient mice. Mononuclear cells were isolated through density gradients centrifugation and then washed twice in phosphate buffered saline (PBS). Detailed clinical characteristics of forty-six B-ALL patients are presented in Table 1.

### B-ALL patient-derived xenografts model

Immunodeficient NOD-scid-IL2Rg-/- (NSI) mice were procured for the experiments.^19^ All mice were bred and housed in specific pathogen-free (SPF) – grade cages and were provided with autoclaved food and water. The protocols were approved by the Institutional Animal Care and Use Committee (IACUC) at Nanfang Hospital. Xenograft-expanded B-ALL cells (BALL#1 and BALL#2) were injected intravenously via the tail-vein into NSI mice aged 8–10 weeks after preconditioning with total body irradiation (TBI, 100 cGy). For drug treatment, Vincristine (Vin, 1 mg/kg) together with Dexamethasone (Dex, 50 mg/kg) were injected intraperitoneally to eight-week-old NSI mice that had been previously transplanted with leukemic cells, with four times per week for four weeks. Phosphate Buffered Saline (PBS) was used as control.To prepare bones for staining, mouse tibiae were initially fixed in formalin for 24 hours, decalcified in EDTA for 2–3 weeks, and then fixed overnight in 4% paraformaldehyde. Subsequently, they were paraffin-embedded, sectioned, stained with H&E, and scanned at 100X magnification.

### Homing assay

Homing assays were performed as described previously.^20^ Briefly, Dex- and saline pre-treated immunodeficiency NSI mice were transplanted with B-ALL cells (1 × 10^6^/mouse) by tail vein injection. 16 hours post-transplantation, BM (femur) was analyzed for the presence of human CD19^+^ B-ALL cells.

### Cell culture

Cells were incubated at 37°C in a humidified atmosphere with 95% air and 5% CO_2_. OP9 stromal cells (CRL-2749) were cultured in α-MEM (HyClone) supplemented with 20% fetal bovine serum (FBS) (Gibco), 2 mM L-glutamine, 1× penicillin/streptomycin. Primary B-ALL cells harvested from xenografts were enriched via magnetic cell sorting using anti-human CD45 MicroBeads (Miltenyi Biotec) and cultured at a density of 1 × 10^6^/ml in RPMI1640 (HyClone, Thermo Scientific), 10% FBS, 2 mM L-glutamine, 1× P/S. For cytokine supplementation, the culture medium was supplemented with 10 ng/ml IGF-1 (Peprotech, 100–11), 10 ng/ml IL-7 (Peprotech, 200–07), 10 ng/ml IL-6 (Peprotech, 200–07). The culture medium was changed every two days. Adipocyte differentiation was induced following a previous study.^21^ Briefly, OP9 cells were cultured as a monolayer. Upon reaching confluence, the cells were maintained for an additional 2 days and then incubated in DMEM supplemented with 10% FBS, 1 µM Dexamethasone, .5 mM isobutyl-methylxanthine, 5 µg/mL insulin, and 1 µM Rosiglitazone (Sigma-Aldrich) for 2 days. Subsequently, the medium was replaced with DMEM plus 10% FBS and 1 µM Dexamethasone for 7–14 days, with fresh medium provided every 2 days.

### Histological analysis

Tissue samples were fixed in 4% formalin, embedded in paraffin, sectioned at 4-μm thickness, and stained with hematoxylin and eosin (H&E). BM biopsies were scanned at 100X magnification. Adipocytes were identified as unstained white and round objects in H&E staining and were automatically counted using Image-Pro Plus 6.0 (Media Cybernetics). The percentage of BM adipocytes was calculated as adipocytes area/total area × 100%.

### Flow cytometry

Flow cytometric analysis was conducted using a Fortessa II instrument (BD Biosciences). In brief, cells were pelleted and resuspended in 50 μl of FACS buffer (2% FBS in PBS) with antibodies, followed by incubation at 4°C for 30 mins in the dark. Peripheral blood, spleen, and BM samples from the mice were processed following standard procedures. FACS data were analyzed using FlowJo software (FLOWJO).

### Apoptosis assays

For apoptosis analysis, cells were stained with the Apoptosis Detection Kit (eBioscience). Cells were washed twice with PBS, suspended in 100 μl of binding buffer containing APC-conjugated Annexin V, and incubated in the dark for 15 min. Subsequently, cells were washed and suspended in 100 μl of binding buffer containing propidium iodide (PI). The percentage of apoptotic cells was quantified through FACS analysis.

### Enzyme-linked immunosorbent assay (ELISA)

Supernatants were collected from the OP9-B-ALL co-culture system and the adipocyte-B-ALL co-culture system after 72 hours. For ELISA assays, equal volumes of supernatants obtained from at least three independent experiments were pooled and subjected to a high-sensitivity ELISA Kit (R&D Systems, Minneapolis, MN, USA) following the manufacturers’ instructions.

### Statistical analysis

Data were expressed as the mean ± standard deviation (S.D.) for a minimum of three independent experiments and were compared using the student t-test. Multiple-group comparisons were performed using One-way analysis of variance (ANOVA), followed by the Bonferroni post-hoc test. Survival was estimated through Kaplan-Meier analysis and compared using the log-rank test. P-values <.05 were considered statistically significant. All statistical analyses were carried out using Prism software, version 7.0 (GraphPad).

## Results

### BM adipocytes increase markedly in B-ALL patients with complete remission

**Figure 1.**
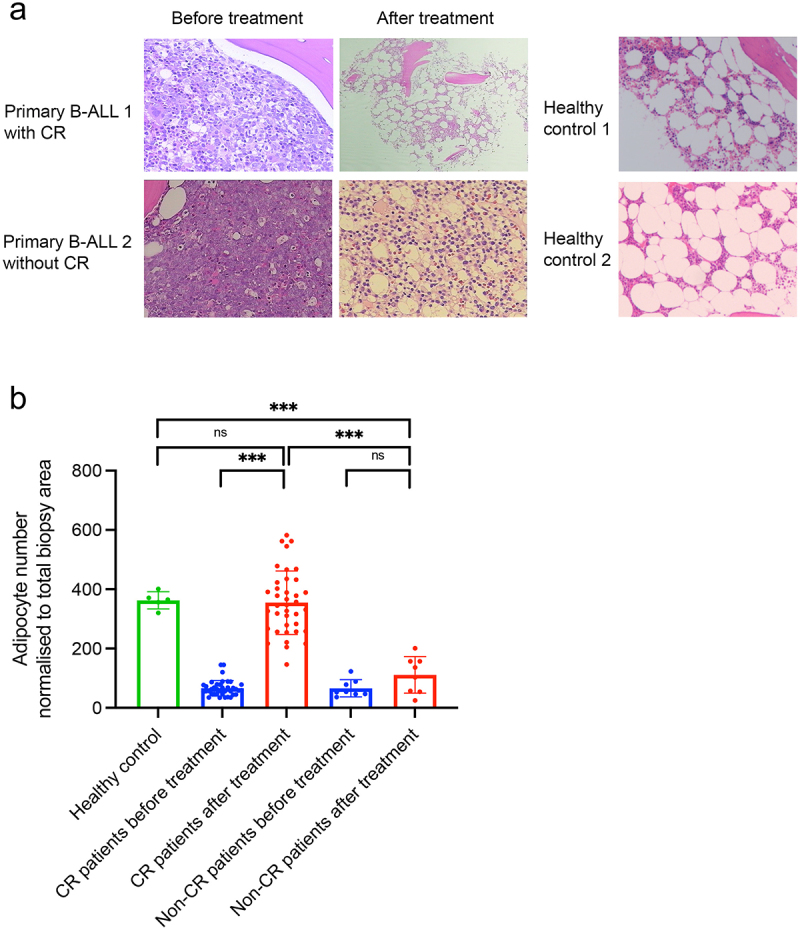
The BM adipocyte niche is dynamically reprogramed during ALL diagnosis and post-treatment. (a) Representative H&E-stained human BM biopsies from completed remission (CR) and non-completed remission (non-CR) B-ALL patients as well as healthy controls. (b) Adipocyte counts in CR or non-CR B-ALL patients as well as healthy controls. Data are presented as the mean ± SD. ***p < .001 by paired T test. N.S means non-significant.

As shown in Figure 1a, at initial diagnosis, BM biopsy of primary B-ALL patient revealed adipocytes were profoundly depleted when compared to healthy controls. However, a striking increase of BM adipocytes were observed after achieving complete remission (CR), which was comparable with that of healthy controls. In contrast, in primary B-ALL patients who did not achieved CR, leukemic cells nearly occupied the entire BM cavity, as presented through hematoxylin and eosin staining. Building on this clinical observation, we hypothesized that the proportion of BM adipocytes could serve as a marker of CR for B-ALL patients. To investigate this, we enrolled 46 paired BM biopsy samples from B-ALL patients taken at the onset of diagnosis and after chemotherapy. Five healthy donors for hematopoietic stem cell transplantation were included as healthy controls. Before chemotherapy, BM biopsy of B-ALL samples exhibited few adipocytes (66 ± 27), but upon achieving CR, BM biopsies demonstrated an sharp increase of BM adipocytes comparable with that of healthy controls (344 ± 66 vs 363 ± 29, *p* = .627), indicating a return of adipocyte population homeostasis upon successful therapy (Figure 1b, Supplemental Table 1).Besides, the percentage of B-ALL cells, identified based on their aberrant immunophenotype or leukemia-associated immunophenotype by multicolor flow cytometry, significantly declined from 62.8 ± 13.7% at diagnosis to 3.03 ± 1.55% after getting CR (*p* < .001). Conversely, patients not achieving complete remission after treatment did not exhibit a substantial increase in adipocyte numbers (Figure 1b).

### Dexamethasone plus Vincristine induce an increase in BM adipocytes in B-ALL patient-derived xenografts

**Figure 2.**
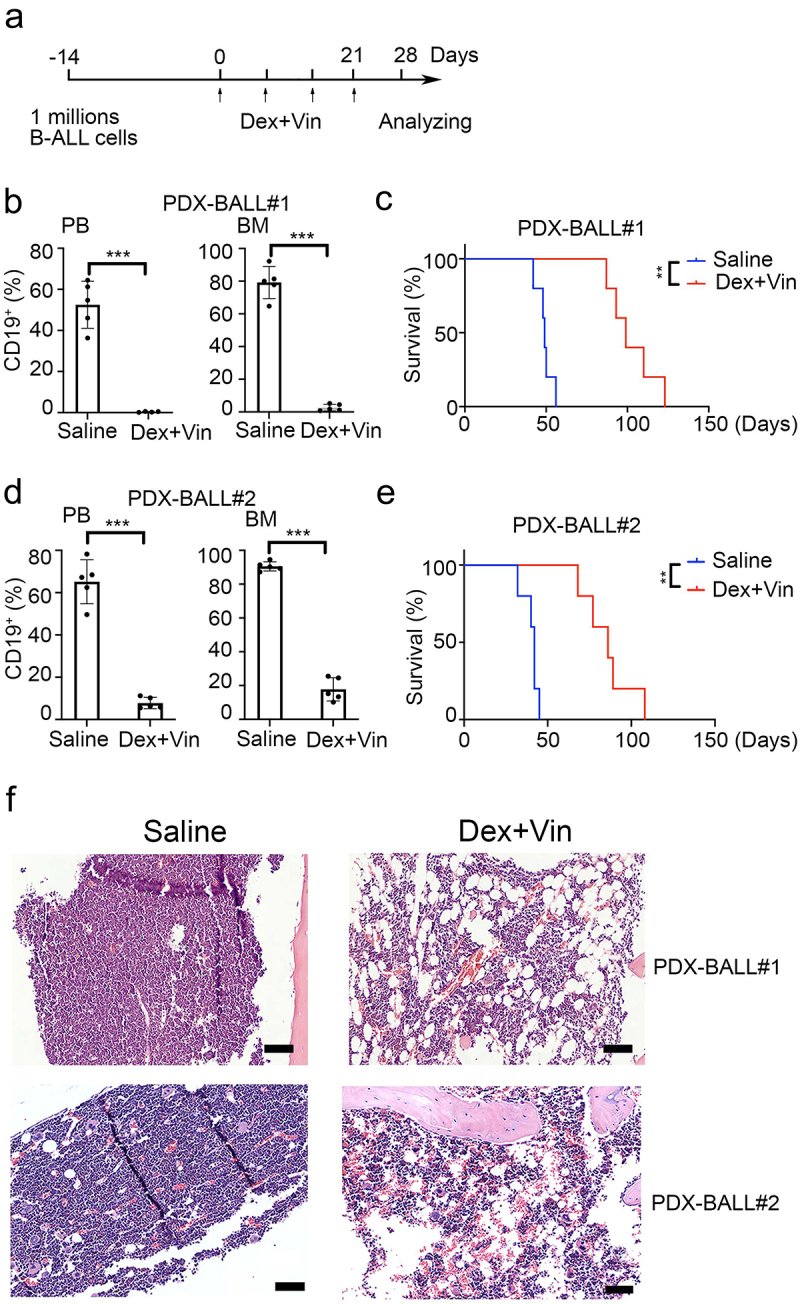
Dexamethasone and vincristine treatment increased BM adipocytes of B-ALL patient-derived xenografts. (a) Experimental procedure. Six-week-old NSI mice were transplanted with B-ALL cells for 2 weeks. B-ALL engrafted mice were treated with dexamethasone (Dex, 50 mg/kg) and vincristine (Vin, 1 mg/kg) for four times every week. Leukemic progressing in mice were analysis at week 4. (b,d) FACS analysis of CD19+% (BALL#1 and BALL#2) in PB and BM of Dex- or Vin-treated and untreated mice. *n* = 5 for each group. ***p < .01 and **p < .001. (c,e) Kaplan-Meier survival curve of xenografted mice (BALL#1 and BALL#2) treated with/without Dex/Vin. *n* = 5 for each group. ***p < .01. (f) Representative H&E-stained femur biopsies from xenografted mice (BALL#1 and BALL#2) treated with/without Dex and Vin. Scale bars = 100 µm.

To investigate whether adipocytes also increased in B-ALL xenograft models, we used samples from two B-ALL patients (third generation) to establish B-ALL patient-derived xenografts. Dexamethasone (Dex) and Vincristine (Vin) were used to replicate clinical treatment, as shown in Figure 2a. After four weeks of Dex plus Vin treatment, the percentage of CD19 positive cells dramatically decreased in the BM of drug-treated mice compared to PBS-treated mice (2.65 ± 2.05% vs 79.22 ± 9.83%, *p* < .001 for PDX-BALL#1 and 17.76 ± 6.86% vs 90.52 ± 2.70%, *p* < .001 for PDX-BALL#2, Figure 2b,d). This effect was similarly observed in peripheral blood (.38 ± .19% vs 52.52 ± 11.48%, *p* = .001 for PDX-BALL#1 and 7.78 ± 2.70% vs 65.16 ± 10.42%, *p* < .001 for PDX-BALL#2). As expected, mice treated with Dex and Vin exhibited extended survival compared to those treated with PBS (median survival, 99 days vs. 49 days, *p* = .009 for PDX-BALL#1 and 86 days vs. 42 days, *p* = .009 for PDX-BALL#2, Figure 2c,e). H&E staining showed that the BM mice treated with Dex and Vin were predominantly filled with adipocytes (Figure 2f). These results indicated that Dex plus Vin treatment substantially increased BM adipocytes in B-ALL patient-derived xenografts, consistent with findings in primary B-ALL patients.

### Increased BM adipocytes retards engraftment of B-ALL cells in PDX models

**Figure 3.**
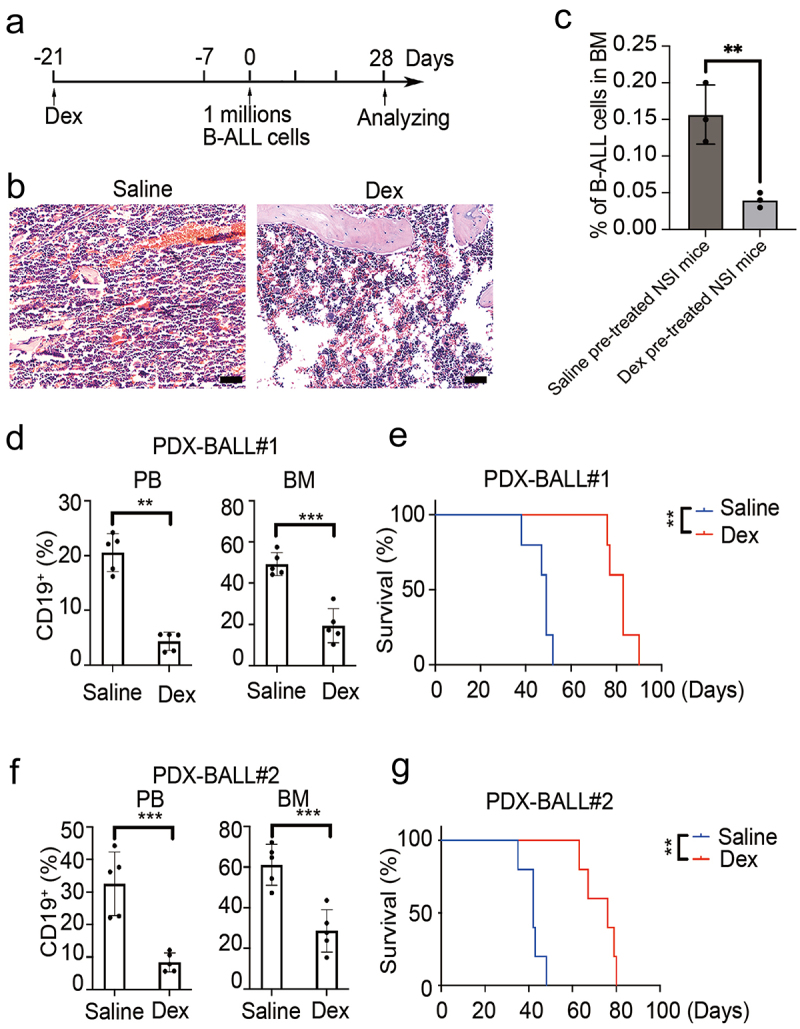
Increased BM adipocyte retard engraftment of B-ALL cells in PDX model. (a) Experimental design. Six-week-old NSI mice were pretreated intraperitoneally with daily dexamethasone (Dex, 50 mg/kg) or saline for two weeks, followed by one week of exposure to primary leukemia cells. Leukemic progressing in mice were analysis at week 4. (b) H&E-stained femur biopsies from mice with/without treated with Dex. Scale bars = 100 µm. (c) Homing assay of B-ALL cells to BM in Dex and saline pre-treated NSI mice. The data are presented as relative homing cells. The error bar indicates the SEM of assays performed in triplicate. (d,f) FACS analysis of the engraftment percentage of leukemic cells (BALL#1 and BALL#2) in PB and BM of Dex-treated and untreated mice (*n* = 5). ***p < .01 and **p < .001. (e,g) Kaplan-Meier survival curve of mice with/without treated with Dex are transplanted B-ALL cells (BALL#1 and BALL#2). *n* = 5 for each group. ***p < .01.

To explore the impact of adipocytic BMM on B-ALL development, mice were pre-treated with Dex to establish an adipocyte-enrichment niche as previously reported.^22^ The procedure is outlined in Figure 3a. Consistent with previous reports, two weeks of Dex pre-treatment significantly increased the proportion of adipocytes in the BM (Figure 3b). One week after Dex withdrawal, both Dex- and saline-treated mice were transplanted with B-ALL cells via tail vein injection. Sixteen hours after transplantation, homing assay of B-ALL cells to BM was conducted. Results showed the percentages of CD19 positive cells in the saline pre-treated mice were significantly higher than that in Dex pre-treated mice (.16 ± .04% vs .04 ± .01%, *p* = .008, Figure 3c). On day 28 post-engraftment, a subset of mice was sacrificed for FACS analysis, while others were kept for survival analysis. As shown in Figure 3d,f, a significantly lower percentage of CD19 positive cells was observed in the BM of Dex-pretreated mice compared to saline pre-treated mice (19.32 ± 8.31% vs 49.26 ± 5.57%, *p* < .001 for PDX-BALL#1 and 28.60 ± 10.43% vs 61.12 ± 10.10%, *p* = .001 for PDX-BALL#2). This result was similarly observed in peripheral blood (4.34 ± 1.68% vs 20.54 ± 3.45%, *p* < .001 for PDX-BALL#1 and 8.36 ± 2.92% vs 32.54 ± 9.78%, *p* = .004 for PDX-BALL#2). Consequently, Dex-pretreated mice exhibited a prolonged overall survival compared to saline-pretreated mice (median survival, 83 days vs 49 days, *p* = .009 for PDX-BALL#1 and 76 days vs 42 days, *p* = .009 for PDX-BALL#2, Figure 3e,g). Thus, our results indicated that increased BM adipocytes might retards engraftment of B-ALL cells in PDX models.

### Adipocytes do not support B-ALL growth *in vitro*

MSCs and adipocytes play crucial roles in the BMM. Previous studies have demonstrated that MSCs can promote the growth of primary B-ALL cells *in vitro*.^23,24^ However, the influence of BM adipocytes on leukemia cells remains controversial and may vary depending on the lineage. Previous studies showed that BM adipocytes negatively affect T-ALL proliferation in vitro and in vivo.^7^ On the contrary, they supported the survival and proliferation of AML blasts from patients.^14^ To further investigate the impact of BM adipocytes on B-ALL cells, we conducted in vitro co-cultures of primary B-ALL cells (leukemic cells from BALL#1 and BALL#2) with adipocytes derived from BM MSCs (OP9 cells) and OP9 stromal cells.

**Figure 4.**
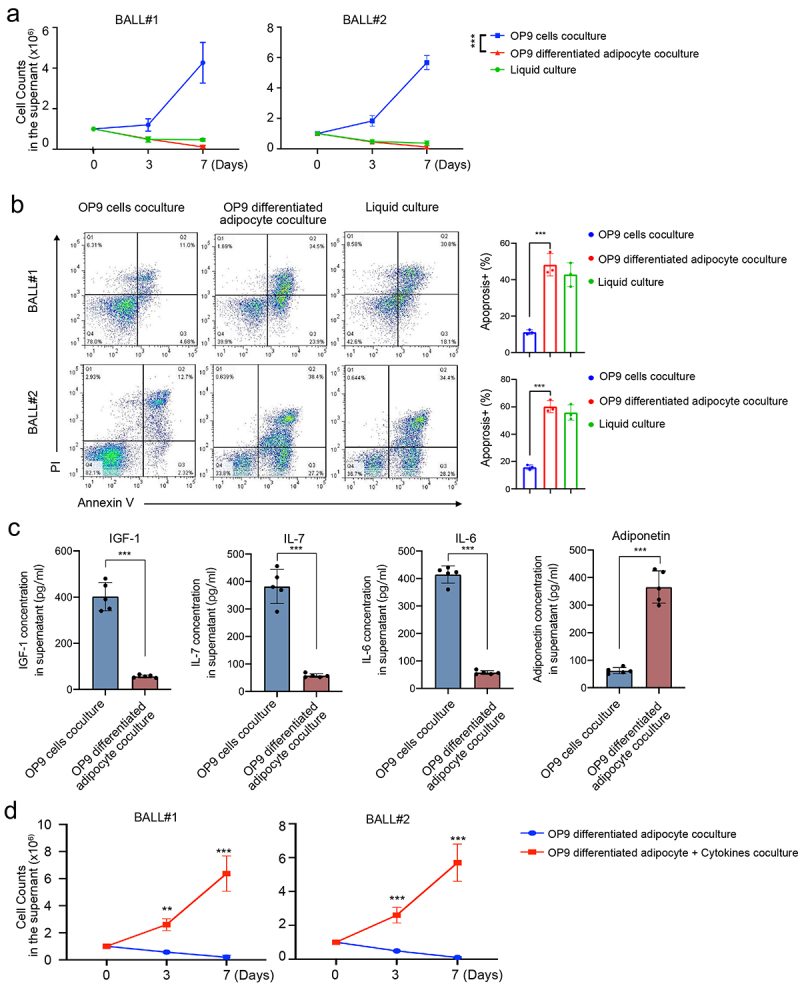
Adipocytes fail to support growth of B-ALL cells in vitro. (a) B-ALL cells (BALL#1 and BALL#2) of one million were seeded in 24-well plates co-culturing with OP9, adipocytes (OP9-derived) or in liquid condition. Cell numbers were counted at day 7. Every 2 days, half of the culture medium was replaced with fresh medium, and viable cell yields were counted with a hemocytometer at day 3 and 7. Data are presented as the mean ± SD. ***p < .001. (b) Apoptosis cells were assayed by annexin V/PI staining at 48 hours. Annexin V+/PI+ were counted as apoptosis cells. (c) Quantification of MSC-secreted cytokines (IGF-1, IL-7 and IL-6) and adipocyte-secreted adiponectin in the stromal cell line OP9 co-culture or adipocyte co-culture system. (d) One million of B-ALL cells (BALL#1 and BALL#2) were co-cultured with adipocytes (OP9-derived) with/without cytokines (IGF1, IL-7 and IL-6, 10 ng/ml each) in 12-well plates in 2 ml of completed RPMI1640. **p < .01 and ***p < .001.

Interestingly, our findings revealed that B-ALL cells co-cultured with adipocytes exhibited poor proliferation, whereas co-culture with OP9 stromal cells significantly enhanced the growth capacity of B-ALL cells (Figure 4a). Furthermore, adipocytes induced higher levels of apoptosis compared to OP9 stromal cells (48.40 ± 5.89% vs 11.50 ± 3.29%, *p* = .001 for PDX-BALL#1 and 48.80 ± 5.86% vs 12.03 ± 3.07%, *p* = .001 for PDX-BALL#2, Figure 4b). To explore the underlying mechanisms, we examined the concentration of MSC-secreted cytokines (IGF-1, IL-7, and IL-6) in the co-culture system, as well as the concentration of adiponectin, a functional marker of BM adipocytes. As depicted in Figure 4c, the OP9 co-culture system exhibited higher concentrations of IGF1, IL-7, and IL-6, while the adipocytes co-culture system showed increased adiponectin levels. Additionally, the addition of cytokines (IGF1, IL-7, and IL-6) to the adipocyte co-culture system effectively alleviated the suppression effect of adipocytes on B-ALL cell growth (Figure 4d). These results collectively suggest that adipocytes do not support the proliferation of B-ALL cells, and this effect may be attributed to the presence of adiponectin secreted by adipocytes themselves and the absence of cytokines typically secreted by MSCs.

## Discussion

In response to chemotherapeutic stress, BMM undergoes significant adaptations.^8,9,25,26^ However, there is a noticeable gap in the literature regarding the dynamic transformation of BMM from the initial disease stage to the post-chemotherapy phase in B-ALL. In the present study, we have shed light on the role of BM adipocytes as a crucial component of the BMM, emerging prominently upon complete remission, which aligns with findings from another report.^27^ Intriguingly, the question arises: why are adipocytes depleted in ALL-BMM? Adipocytes originate from mesenchymal stem cells (MSCs) with multilineage potential.^28,29^ A previous study suggested that the ALL disease process disrupts the regenerative growth and the differentiation of MSCs, resulting in a decreased number and blocked differentiation of MSC precursors within the ALL-affected BM.^27^ This may elucidate the disease-associated loss of adipocytes. However, this effect appears reversible upon ALL clearance. As corroborated in our study, leukemia burden was eradicated in the peripheral blood and BM in B-ALL patient-derived xenografts treated with Dexamethasone (Dex) plus Vincristine (Vin), concomitant with a noticeable increase in the proportion of BM adipocytes. Similar observations were made in ALL patients who achieved complete remission after treatment.

While it is generally accepted that the BM adipocytic niche contributes to leukemia development and progression,^30,31^ recent studies have presented opposing arguments, suggesting that BM adipocytes may have a negative impact on leukemia progression.^7,18,32^ For instance, Tucci J et al. reported ALL cells stimulate the release of free fatty acids (FFA) from adjacent adipocytes. Adipocyte-derived FFA, particularly unsaturated FFA, is absorbed by the ALL cells and integrated into cell membranes and lipid droplets, serving as a metabolic fuel and contributing to modest chemotherapy resistance.^31^ Conversely, other studies have indicated that BM adipocytes inhibit ALL development. Lu et al.^18^ demonstrated that fasting, a condition known to enhance marrow adipose tissue, inhibited the engraftment and progression of B-ALL and T-ALL in various mouse model systems. This suggests that adipocytes act as “enemies” of leukemia cells within the BMM. Mechanistically, they found that fasting reduced circulating and local leptin levels but upregulated the expression of the leptin receptor in leukemia cells, activating downstream signaling pathways as the primary cause of inhibiting leukemia initiation and progression.

Additionally, Heydt et al.^27^ reported adipocytes significantly impaired the growth capacity of independent ALL cell lines in vitro co-cultures. Their study also confirmed the limited expansion of ALL cells in vivo within adipocyte-rich niches. Consistently, our results reveal that adipocytes inhibit ALL growth in vitro, even in the absence of drug exposure. Furthermore, B-ALL patient-derived xenografts demonstrated restricted homing of B-ALL cells to BM in the context of an adipocyte-enriched BMM.

However, the findings from Heydt Q et al.^27^ present a contrasting perspective, suggesting that adipocyte niches do not significantly induce apoptosis but instead hinder cell cycle progression. This results in the consistent expansion of a quiescent cell population, accompanied by variable reductions in S/G2/M progression. Consequently, they concluded that adipocytes increase the quiescence of ALL cells and their resistance to multiple stresses, which could potentially contribute to leukemia resurgence. Conversely, our study provides additional insight into the impact of adipocyte niches on apoptosis induction.

Much like MSC, which functions as endocrine tissues secreting various cytokines, BM adipocytes also possess the capability to synthesize and release adipocytokines, such as adiponectin. Adiponectin has been shown to significantly inhibit the progression of ALL under the regulation of prostaglandin synthesis.^33^ In contrast, MSCs in the leukemic BMM promote the growth of leukemia cells by secreting a series of cytokines (e.g., IGF-1, IL-7, and IL-6).^34–37^ Indeed, we observed a higher concentration of IGF1, IL-7, and IL-6 in the OP9 co-culture system, while adiponectin levels were elevated in the adipocyte co-culture system. Furthermore, the addition of cytokines (IGF1, IL-7, and IL-6) to the adipocyte co-culture system markedly reversed the suppressive effect of adipocytes on the growth of B-ALL cells. Therefore, in our opinion, the proliferation arrest of B-ALL cells within an adipocyte-enriched niche after disease remission can be attributed to the presence of adiponectin and the absence of cytokines secreted by MSCs.

## Conclusions

Our findings highlight the dynamic transformation of the BM adipocytic niche during the initiation and complete remission of B-ALL, along with its impact on B-ALL cells. Functionally, the reestablishment of an adipocyte-rich BMM leads to a shift in the fate of ALL cells, resulting in proliferation inhibition and apoptosis induction. Mechanistically, this effect may be attributed to the presence of adiponectin secreted by adipocytes themselves and the absence of cytokines secreted by MSCs.

## List of abbreviations

**Table:** 

BMM	bone marrow microenvironment
T-ALL	T cell acute lymphoblastic leukemia
B-ALL	B cell acute lymphoblastic leukemia
MSC	mesenchymal stem cell
BM	bone marrow
PB	peripheral blood
PDX	patient derived xenografts
FBS	fetal bovine serum
DMSO	dimethyl sulfoxide
NSI	NOD-scid-IL2Rg-/-
JAK/STAT	Janus kinase – signal transducer and activator of transcription
MAPK	mitogen-activated protein kinasePI3K: phosphatidylinositol-3 kinase
Dex	Dexamethasone
Vin	Vincristine
P/S	penicillin/streptomycin

## Supplementary Material

Supplemental Table 1.docx
