# Supplementary material for: Dynamic evolution of bone marrow adipocyte in B cell acute lymphoblastic leukemia: insights from diagnosis to post-chemotherapy
Source: Cancer Biol Ther. 2024 Mar 11;25(1):2323765. doi: 10.1080/15384047.2024.2323765 (PMC10936623; doi:10.1080/15384047.2024.2323765)
Supplement: Supplemental Table 1.docx [file KCBT_A_2323765_SM1945.docx]

**Supplemental Table 1.** The percentage and absolute value of BM adipocytes in B-ALL.

| **ID** | **Gender** | **Age** | **Adipocytes (%) and numbers in BM biopsy^1^** | | **Adipocytes (counts) in BM biopsy^1^** | | **Response^2^** | **Follow up (Days)** |
| --- | --- | --- | --- | --- | --- | --- | --- | --- |
|  |  |  | **Before treatment** | **After treatment** | **Before treatment** | **After treatment** |  |  |
| NF001 | Female | 44 | 10 | 60 | 63 | 389 | CR | 892 |
| NF002 | Female | 41 | 5 | 40 | 87 | 283 | CR | 241 |
| NF003 | Male | 28 | 10 | 80 | 120 | 478 | CR | 215 |
| NF004 | Male | 23 | 10 | 70 | 56 | 378 | CR | 183 |
| NF005 | Female | 30 | 10 | 60 | 79 | 366 | CR | 243 |
| NF006 | Female | 53 | 10 | 5 | 53 | 54 | NR | 92 |
| NF007 | Female | 22 | 15 | 60 | 47 | 254 | CR | 755 |
| NF008 | Male | 64 | 5 | 60 | 77 | 378 | CR | 398 |
| NF009 | Female | 41 | 3 | 50 | 57 | 326 | CR | 118 |
| NF010 | Male | 16 | 5 | 40 | 58 | 267 | CR | 122 |
| NF011 | Female | 53 | 5 | 90 | 54 | 362 | CR | 125 |
| NF012 | Male | 18 | 3 | 65 | 35 | 357 | CR | 165 |
| NF013 | Male | 25 | 5 | 40 | 35 | 257 | CR | 123 |
| NF014 | Male | 24 | 3 | 70 | 78 | 391 | CR | 165 |
| NF015 | Female | 38 | 10 | 80 | 89 | 402 | CR | 59 |
| NF016 | Female | 53 | 20 | 60 | 46 | 331 | CR | 149 |
| NF017 | Female | 39 | 10 | 50 | 78 | 258 | CR | 48 |
| NF018 | Male | 21 | 5 | 40 | 63 | 321 | CR | 359 |
| NF019 | Male | 15 | 5 | 75 | 36 | 432 | CR | 129 |
| NF020 | Male | 24 | 5 | 50 | 44 | 279 | CR | 138 |
| NF021 | Male | 17 | 2 | 10 | 36 | 157 | NR | 94 |
| NF022 | Female | 46 | 5 | 15 | 47 | 201 | NR | 60 |
| NF023 | Male | 38 | 4 | 5 | 46 | 25 | NR | 45 |
| NF024 | Male | 22 | 2 | 55 | 35 | 316 | CR | 203 |
| NF025 | Female | 58 | 5 | 50 | 56 | 316 | CR | 85 |
| NF026 | Male | 19 | 3 | 20 | 54 | 136 | NR | 66 |
| NF027 | Male | 39 | 5 | 10 | 67 | 246 | CR | 175 |
| NF028 | Male | 29 | 15 | 79 | 68 | 423 | CR | 98 |
| NF029 | Female | 33 | 10 | 60 | 59 | 327 | CR | 243 |
| NF030 | Male | 39 | 5 | 5 | 78 | 103 | NR | 63 |
| NF031 | Male | 31 | 5 | 50 | 67 | 347 | CR | 158 |
| NF032 | Male | 31 | 5 | 60 | 89 | 389 | CR | 61 |
| NF033 | Male | 57 | 15 | 80 | 145 | 345 | CR | 63 |
| NF034 | Male | 23 | 5 | 80 | 46 | 362 | CR | 88 |
| NF035 | Male | 24 | 5 | 80 | 68 | 435 | CR | 382 |
| NF036 | Female | 28 | 10 | 40 | 88 | 256 | CR | 634 |
| NF037 | Male | 37 | 20 | 50 | 145 | 318 | CR | 70 |
| NF038 | Male | 31 | 5 | 90 | 56 | 382 | CR | 537 |
| NF039 | Male | 56 | 1 | 70 | 63 | 466 | CR | 127 |
| NF040 | Female | 59 | 2 | 30 | 72 | 311 | CR | 87 |
| NF041 | Female | 39 | 5 | 70 | 45 | 345 | CR | 183 |
| NF042 | Female | 32 | 10 | 10 | 123 | 157 | NR | 65 |
| NF043 | Male | 15 | 5 | 30 | 34 | 305 | CR | 65 |
| NF044 | Female | 22 | 5 | 80 | 47 | 217 | CR | 62 |
| NF045 | Female | 54 | 10 | 2 | 89 | 57 | NR | 65 |
| NF046 | Male | 15 | 30 | 70 | 57 | 468 | CR | 126 |

1. BM sections were scanned at 100X magnification. Adipocytes were identified as unstained white and round objects in hematoxylin and eosin (H&E) staining sections and were automatedly counted by Image-Pro Plus 6.0 (Media Cybernetics). Percentage of BM adipocytes was calculated as adipocytes area / total area x 100%.
2. ^2^CR: completed remission. NR: non-remission.
